# Computer-aided detection thresholds can guide repeat rapid molecular testing in TB screening

**DOI:** 10.5588/ijtldopen.25.0830

**Published:** 2026-06-15

**Authors:** B. Datta, A. Story, E. Rogers, S. Kapoor, R. Raju, P. Goyal, A. Prakash, J.P. Tripathy, A. Jaiswal, A. Hayward, R. Guleria, N. Trehan

**Affiliations:** 1 Department of Respiratory and Sleep Medicine, Medanta-The Medicity, Gurgaon, India;; 2 University College London, London, UK;; 3 Institute for Global Health, University College London, London, UK;; 4 District Tuberculosis Office, District Karnal, India;; 5 State Tuberculosis Office, Panchkula, India;; 6 Department of Public Health and Community Medicine, Tufts University School of Medicine, Boston, MA, USA;; 7 Department of Community Medicine, All India Institute of Medical Sciences, Nagpur, India;; 8 Medanta-The Medicity, Gurgaon, India.

**Keywords:** computer-assisted detection, point-of-care testing, active case finding, molecular testing

Dear Editor,

The introduction of WHO-approved molecular rapid diagnostic tests (mWRD) has substantially increased bacteriological confirmation, improved detection of drug resistance, and shortened time to diagnosis.^1,2^ WHO recommends universal access to mWRD.^3^ While initial mWRD testing is being scaled up globally, a second mWRD can increase case detection in smear-negative presumptive TB patients, but at a substantial additional cost.^4^ WHO recommends considering repeat testing in children in settings with a high pretest probability and in adults with an initial negative result after clinical re-evaluation.^2^ Evidence to guide repeat mWRD testing among adults across diverse settings remains limited.

Community-based active case finding (ACF), increasingly adopted by national TB programmes, represents a critical context in which this evidence gap is particularly relevant. ACF screening strategies are increasingly using portable chest X-ray (CXR) and computer-aided detection (CAD) software, which has been shown to outperform human readers in triaging presumptive pulmonary TB and reduce mWRD cartridge use by 50%.^5–7^ An initial negative mWRD among individuals with strong clinic-radiological suspicion presents a diagnostic dilemma in ACF settings, where early, paucibacillary disease is common.^6,8^ Missing the diagnosis risks progression and transmission, while empiric treatment without confirmation risks overtreatment and drug resistance.^9,10^ The value of repeat mWRD in community ACF for people with a high clinico-radiological suspicion of TB following an initial negative mWRD has, to our knowledge, not been studied. Here, we assessed CAD-guided thresholds to triage mWRD-negative individuals for repeat testing, examined associated costs, and compared universal and CAD-guided testing strategies in terms of yield, efficiency, sensitivity gains, and cost.

This nested, non-matched cohort study was embedded within a cluster-randomised controlled trial (c-RCT) of a community ACF programme in rural Karnal, Haryana, India. The study population included all presumptive TB cases with TB compatible radiological changes and who had an initial negative mWRD test result. The study team, including local community health workers, conducted door-to-door surveys. Eligible participants were presumptive pulmonary TB cases (presenting with any of the symptoms: cough > 2 weeks, fever > 2 weeks, unexplained weight loss, haemoptysis), unscreened household contacts of recently diagnosed TB cases, and recent treatment defaulters. Identified participants were invited to local screening camps for demographic and clinical data collection followed by digital CXR. Radiographs were interpreted on-site using CAD (QureAI qXR 3.2) with a threshold of ≥0.6 (this cut-off is supported by evidence in the literature and the manufacturer’s default threshold for triaging) to triage for first mWRD testing.^11^ CAD reporting was augmented by expert radiological and clinical opinion provided either on-site or reporting synchronously or asynchronously using teleradiology. Sputum samples were assessed for quality, with Xpert MTB/RIF results available within 24 h. Participants with negative initial mWRD results were invited for a second mWRD within 4 weeks. All positives were notified, linked to treatment, offered HIV testing, and household contacts invited for screening. Data were captured electronically on secure tablets and included demographics, clinical variables, digital X-ray results, CAD score, and first and second mWRD test results. Anonymised data were extracted from the trial database and analysed in Stata MP 18.5. Patient flow was presented in a flowchart, and 2-second mWRD testing strategies – universal and CAD-guided – were compared for sensitivity, yield, number needed to diagnose, and cost per positive detected. The study was approved by the Medanta Institutional Ethics Committee (IEC 1405/2022). Written informed consent was obtained from all participants.

All 6,673 presumptive pulmonary TB underwent CXR, 29.8% (1,989/6,673) had radiological changes suggestive of TB. The majority (72.3%, 1,438/1,989) had a CAD Score ≥ 0.6, and the remaining 27.7% were triaged for mWRD based on clinical opinion. 84.4% (n = 1,678/1,989) underwent first mWRD test, of whom 13.0% (n = 218/1,678) tested positive. The remaining 1,460 negatives were offered repeat testing, of whom 711 (48.7%) underwent second mWRD test. 2.4% (n = 17/711) of those who underwent a second mWRD test were positive. All second mWRD-positive results had a CAD score > 0.85 (17/17, 100%), and 94% (16/17) had a CAD score ≥ 0.9 (see Figure).

**Figure.**
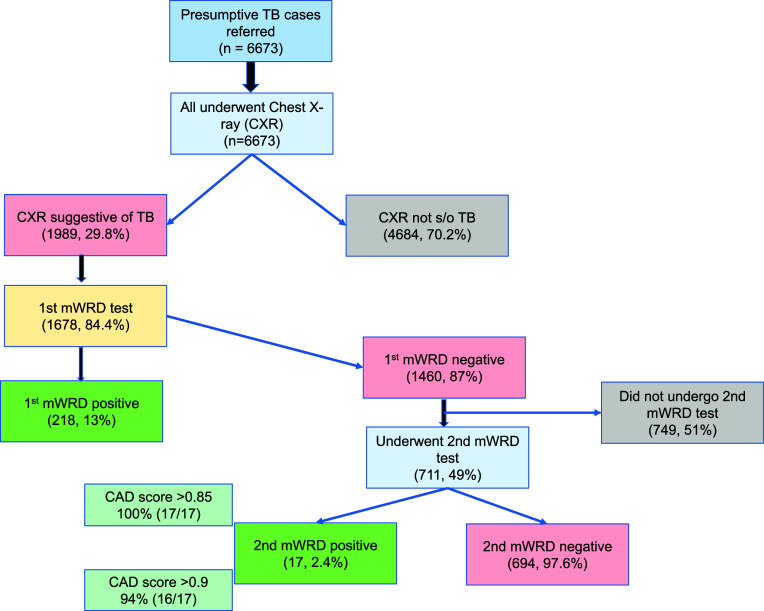
Flow of study participants through the testing algorithm. mWRD = molecular WHO-approved rapid diagnostic tests; CAD = computer-aided detection.

With a CAD cut-off of 0.9 to triage into second mWRD, the proportion of individuals with a positive second mWRD test was significantly higher (16/364, 4.4%) compared to a score < 0.9 (1/347, 0.29%, *P* value <0.001). With a CAD threshold ≥ 0.9, only 347 (49% fewer tests) would require testing, detecting 16/17 mWRD-positive cases (sensitivity: 94.1%) compared to universal testing. The targeted approach improved testing yield to 4.61% (92.9% higher yield than universal testing: 4.61% vs. 2.39%). At a unit cost of USD 10 per test, the total testing cost decreased from USD 7,110 to USD 3,470, with the cost per positive detected nearly halved (USD 217 vs. USD 418) – see Table.

**Table.** Comparison of mWRD testing strategies: universal and CAD score–targeted (CAD score ≥ 0.9).

Metric	Universal testing	CAD score–guided testing (CAD score ≥ 0.9)
Total individuals tested	711	347
mWRD positives detected	17	16
Sensitivity (% of total positives)	100.0% (17/17)	94.1% (16/17)
Specificity	100%	100%
Cases missed	0	1
Tests performed	711	347
Tests saved versus universal	–	51.2% fewer
Positives per 100 tests (yield)	2.39	4.61
Yield improvement versus universal	–	+92.9%
Number needed to diagnose	41.82	21.69
Cost per test (USD)	10	10
Total testing cost (USD)	7,110	3,470
Cost per positive detected (USD)	418.24	216.88

Use of CAD thresholds to inform targeted repeat mWRD testing among individuals in community ACF programmes who have an initial negative mWRD test can substantially improve diagnostic yield, efficiency, and cost-effectiveness while maintaining high sensitivity. To our knowledge, these findings are novel and relevant to community ACF programmes internationally.

Previous studies have confirmed overtreatment, higher mortality, and missed other diagnosis with empiric therapy.^10,12,13^ Studies that have cultured sputum of patients started on empiric treatment have found a 10% positivity rate, indicating that ∼90% patients are true negatives, receiving unwarranted treatment, driving the emergence of drug resistance.^14^ In this context, this study highlights the potential for CAD to function as a triage tool, in the absence of a human reader, using optimised cut-offs to direct patients to repeat mWRD testing and thereby improve diagnostic opportunities for those who might otherwise receive empirical or no treatment at all.

A highly homogeneous, stable population with a low prevalence of HIV necessitates further studies to extrapolate our findings to diverse settings. The ACF activity was organised and well executed by an experienced research team supported by local health workers, as demonstrated by the very high rates of screening camp attendance and uptake of digital CXR and initial mWRD test. Uptake of second mWRD test was 49%, raising potential selection bias; however, those retested were broadly similar to those not retested, differing mainly in age, prior TB history, and CAD score. Our study team rigorously quality controlled samples for mWRD testing to minimise false negative results due to poor quality specimens. Despite these efforts, community ACF programmes will reach a higher proportion of individuals with paucibacillary disease in whom bacterial DNA might be below the detection limit for any mWRD, wherein alternative sampling strategies, combined with higher sensitivity tests are likely needed.^15,16^

Using CAD thresholds to guide repeat mWRD testing in community ACF programmes has the potential to reduce costs while improving diagnostic yield without compromising sensitivity. Further studies are needed to assess its effectiveness, cost-effectiveness, and public health impact across diverse settings.
